# The Brazilian Society of Dermatology on the centennial of the Anais Brasileiros de Dermatologia^☆^

**DOI:** 10.1016/j.abd.2024.09.001

**Published:** 2024-10-28

**Authors:** Heitor de Sá Gonçalves, Carlos Baptista Barcaui, Francisca Regina Oliveira Carneiro, Márcio Soares Serra, Rosana Lazzarini, Fabiane Andrade Mulinari Brenner

**Affiliations:** a Centro de Referência Nacional em Dermatologia Sanitária Dona Libânia, Secretaria da Saúde, Governo do Estado do Ceará, Fortaleza, CE, Brazil; b Instituto de Dermatologia Prof. Rubem David Azulay, Santa Casa da Misericórdia do Rio de Janeiro, Rio de Janeiro, RJ, Brazil; c Department of Dermatology, Universidade do Estado do Pará, Belém, PA, Brazil; d Private Practice, RJ, Rio de Janeiro, Brazil; e Dermatology Clinic, Irmandade Santa Casa de Misericórdia de São Paulo, São Paulo, SP, Brazil; f Service of Dermatology, Hospital de Clínicas, Universidade Federal do Paraná, Curitiba, PR, Brazil

It was the year 1925, during which many events in Brazilian economic, political and social life took place. The world was going through a period of transition and reconstruction after World War I (1914-1918). Many European countries were still recovering from the devastating effects of the war and economies were trying to stabilize and rebuild, and there was a widespread desire for stability and peace. In Brazil, the first national automobile factory, General Motors, was opened in São Paulo. In that same year, Irineu Marinho founded *O Globo*, a daily newspaper, and the first *São Silvestre* Race was held. At the same time, thirteen years after the founding of the Brazilian Society of Dermatology (SBD), Eduardo Rabello was responsible for the first issue of *Anais Brasileiros de Dermatologia*, still under Fernando Terra administration at SBD.

When we leaf through the first issue of *Annaes Brasileiros de Dermatologia e Syphilographia,* dated January 1925, kept in the largest Dermatology library in South America, at SBD headquarters in Rio de Janeiro; in addition to the peculiar smell of very old paper, it is possible to feel the enthusiasm with which Eduardo Rabello, faithful to the French Dermatology, decided to create a larger publication with a scientific purpose.^1^

Although it has always been a journal under the scientific supervision of the Brazilian Society of Dermatology, curiously, during its first 22 years of existence, *Anais Brasileiros de Dermatologia* was owned by individuals, and its first owner was Oscar Silva Araújo. It was only on October 10, 1947, that Antônio Fernandes da Costa Júnior, then President of SBD transferred the rights of *Anais Brasileiros de Dermatologia* to the assets of our society, according to the deed signed on that occasion at the 16th Notary Office of the City of Rio de Janeiro.^2^

With the exception of the year 1931, when the journal was not published due to financial constraints for the construction of *Pavilhão São Miguel*, *Anais Brasileiros de Dermatologia* has reached its centennial without publication interruption. A World War, the Cold War, the landing on the moon, the fall of the Berlin Wall, pandemics, and 21 years of military government in Brazil, none of these prevented our journal from being published, gradually gaining worldwide scientific relevance.

On behalf of the SBD, we would like to thank each Chief Editor and their editorial teams since the founding of ABD for the brilliant work they have performed, as well as all the authors who submitted their scientific works to the scrutiny of our Society. We recently achieved the highest Impact Factor ever achieved by a Dermatology journal in South America. This is the result of the consistent work carried out throughout this century of existence.

In the current context, the challenge remains to keep our journal relevant to the scientific community. Our greatest vocation in Brazil has always been issuing publications related to infectious and parasitic diseases, a vanguard that we can never forget. On the other hand, the number of international articles that are submitted is ever-increasing. By connecting these two realities, we have everything we need to increase the international competitiveness of ABD, always seeking excellence and honoring all those who preceded us in the running of this journal over the last one hundred years.

## Financial support

None declared.

## Authors' contributions

Heitor de Sá Gonçalves: Approval of the final version of the manuscript; drafting and editing of the manuscript.

Carlos Baptista Barcaui: Approval of the final version of the manuscript; drafting and editing of the manuscript.

Francisca Regina Oliveira Carneiro: Approval of the final version of the manuscript; drafting and editing of the manuscript.

Márcio Soares Serra: Approval of the final version of the manuscript; drafting and editing the manuscript.

Rosana Lazzarini: Approval of the final version of the manuscript; drafting and editing of the manuscript.

Fabiane Andrade Mulinari Brenner: Approval of the final version of the manuscript; drafting and editing of the manuscript.

## Conflicts of interest

None declared.
